# Simple, Fast and Sensitive Voltammetric Procedure for Copper Ion Determination Using a Solid Gold Microelectrode Array

**DOI:** 10.3390/s26134305

**Published:** 2026-07-07

**Authors:** Mieczyslaw Korolczuk, Mateusz Ochab, Iwona Gęca

**Affiliations:** Institute of Chemical Sciences, Faculty of Chemistry, Maria Curie Sklodowska University, 20-031 Lublin, Poland; mieczyslaw.korolczuk@mail.umcs.pl (M.K.); mateusz.ochab@mail.umcs.pl (M.O.)

**Keywords:** copper, gold microelectrode array, determination, anodic stripping voltammetry

## Abstract

The present study reports the application of a gold microelectrode array to determine copper(II) ions by anodic stripping voltammetry (ASV). The microelectrode characterization of the presented working electrode was investigated. Moreover, the way of its preparation ensures its reusability and eco-friendly character, thanks to the use of environmentally benign electrode material. The procedure does not require modification of the surface of the working electrode. Main experimental parameters were optimized, including pH and a concentration of the supporting electrolyte, activation and deposition conditions, and square wave parameters. The calibration graph was linear in the range of Cu(II) concentrations from 2 × 10^−9^ to 2 × 10^−7^ mol L^−1^ (with a deposition time of 30 s) and from 5 × 10^−10^ to 5 × 10^−8^ mol L^−1^ (with a deposition time of 90 s; RSD was 4.7% (*n* = 3) for a 1 × 10^−8^ mol L^−1^ of Cu(II)). The limit of detection was equal to 1.93 × 10^−10^ mol L^−1^ (t_acc_ = 90 s). The correctness of the developed procedure was successfully checked by analysis of certified reference material and a tap water sample, confirming the possibility of its practical application. Satisfactory recovery values were also obtained during the analysis of an environmental water sample.

## 1. Introduction

The determination of copper(II) ions in environmental water matrices, including rivers, lakes, and groundwater, is a fundamental component of ecological and sanitary monitoring. Although copper is an essential micronutrient for living organisms [1,2], the margin between its physiological requirement and its ecotoxicity is remarkably narrow [3]. The presence of copper in water often indicates specific sources of anthropogenic contamination like effluents from electroplating plants, copper mines, metallurgy, and electronics manufacturing; the application of copper-based pesticides and fungicides; and corrosion of copper piping within water distribution systems [4,5]. Copper is not biodegradable. It accumulates in bottom sediments and in the tissues of living organisms, which facilitates bioaccumulation that causes animals at higher levels of the food ladder to be at risk of ingesting dietary sources with significantly elevated concentrations of the metal [6,7].

To date, copper ions have frequently been determined by means of several electrochemical techniques [8,9,10,11]. Among them, stripping voltammetry is often chosen for its competitive detection limits and relatively inexpensive equipment. For this purpose, a variety of working electrodes were utilized. These included hanging mercury drop and mercury film electrodes [12,13,14,15], gold and modified gold electrodes [16,17,18,19,20], electrodes modified with gold nanoparticles [21], various carbon-based electrodes [22,23,24,25,26,27,28,29], metal (bismuth, antimony or lead) film electrodes [30,31,32,33], a carbon paste electrode modified with platinum nanoparticles [34], a glassy carbon electrode modified with ferrocenyl crown ether [35], an antimony trioxide-modified multi-walled carbon nanotube paste electrode [36], and an array of iridium ultramicroelectrodes [37]. Nevertheless, the practical utility of several aforementioned electrodes is constrained either by the toxicity of their constituent materials or by protracted, multi-step fabrication protocols. To evaluate the efficacy of the proposed voltammetric method, its analytical performance is benchmarked against previously reported procedures utilizing diverse working electrodes, as detailed in Table 1.

The research described below demonstrates the application of a previously proposed [38] solid gold microelectrode array for square wave anodic stripping voltammetric determination of copper ions. While individual microelectrodes suffer from low intensity of the recorded current, the application of microelectrode arrays or ensembles addresses this deficit. By summing responses from these platforms, an enhanced total current is achieved [39], which is resistant to electrical interference. The important advantage of the presented procedure is the fact that no surface modification was used, and so, the presented protocol is simple, relatively short and environmentally friendly. The obtained detection limit for copper ion determination was 1.93 × 10^−10^ mol L^−1^ for a deposition time of 90 s. The obtained results and the literature data presented in Table 1 demonstrate that only the application of modified electrodes yielded lower limits of detection than the proposed procedure. While a study employing the gold microelectrode achieved a similar detection limit, it involved a deposition time over twice as long, thereby leading to the conclusion that the developed protocol is more straightforward and less time-consuming. The presented procedure was applied for the copper ion determination in the certified reference material TM 26.5. The obtained results of the certified reference material analysis confirmed the accuracy of the developed voltammetric procedure. The developed procedure was also used for analysis of a tap water sample, obtaining results consistent with those of the analysis performed using the ICP-MS as a comparative method. Moreover, after the analysis of environmental water samples, excellent recovery values were yielded, demonstrating the reliability of the developed procedure. The developed analytical procedure extends the application range of this type of working electrodes.

## 2. Materials and Methods

### 2.1. Apparatus

Voltammetric investigations were performed using a μAutolab potentiostat PGSTAT 128N (Eco Chemie, Utrecht, The Netherlands) controlled via GPES 4.9 software and configured with a conventional three-electrode electrochemical cell (10 mL). The experimental setup employed a gold microelectrode array as the working electrode, a platinum wire auxiliary electrode, and a Ag/AgCl/NaCl reference electrode. Detailed procedures regarding the fabrication of the gold microelectrode array have been comprehensively described in a prior publication [38]. The way it is constructed leads to obtaining the electrode with long reusability and high stability. Prior to daily measurements, the surface of the gold microelectrode array was polished using 1500- and 2500-grit sandpapers, rinsed with deionized water, and sonicated in an ultrasonic bath for 30 s. Optical characterization of the array was performed using an MA200 inverted metallographic microscope (Nikon, Shinagawa, Japan). Morphological evaluation was conducted via scanning electron microscopy (SEM) using a TESCAN Vega 3LMU instrument (Tescan Group, a.s., Brno–Kohoutovice, Czech Republic) operated at an acceleration voltage of 20 kV in secondary electron mode. Energy-dispersive X-ray spectroscopy (EDX) analysis was carried out using an integrated AZtecEnergy advanced microanalyzer (Oxford Instruments, High Wycombe, UK). Inductively coupled plasma mass spectrometric analysis was performed using ICP-MS XSERIES-II (Thermo Scientific, Bremen, Germany) with a quartz pneumatic nebulizer and a glass spray chamber. The final operating conditions were as follows: radio frequency power of 1400 W, plasma gas, auxiliary gas, and nebulizer gas flow rates of 13 L min^−1^, 0.95 L min^−1^, and 0.7 L min ^−1^, respectively. The number of replicates was 3. The certified reference material EnviroMAT Ground Water (Low ES-L-2 and High ES-H-2) was used for daily quality control verification of the ICP-MS analytical technique.

### 2.2. Reagents

All chemicals utilized in this study were of analytical reagent or Suprapur grade. Deionized water purified via a Milli–Q system was employed for the preparation of all solutions. A 1 mol L^−1^ acetate buffer (pH 3.3) was prepared from CH_3_COOH and NaOH purchased from Merck (Rahway, NJ, USA). A standard stock solution of Cu(II) at a concentration of 1 g L^−1^ was sourced from Fluka (Buchs, Switzerland). Working solutions of Cu(II) at a concentration of 1 × 10^−5^ mol L^−1^ were prepared daily by appropriate dilution of the stock solution with 0.01 mol L^−1^ HNO_3_. Interfering ion standard solutions at a concentration of 1 g L^−1^ were obtained from Fluka (Buchs, Switzerland). For method validation, certified reference material TM 26.5 (Environment and Climate Change, Gatineau, QC, Canada) was employed. Humic acid sodium salt (HA) and river fulvic acid (FA) were purchased from Aldrich (St. Louis, MO, USA) and from the International Humic Substances Society (Denver, CO, USA), respectively.

### 2.3. Sample Preparation

A real water sample, collected from the Bystrzyca River, was initially filtered through a 0.45 μm membrane filter and acidified to pH 1.5 by the addition of 1 mol L^−1^ HNO_3_. To decompose organic matter, the sample underwent UV irradiation mineralization for 3 h. Subsequently, the sample was stored in polypropylene bottles at 4 °C prior to voltammetric analysis. Before the voltammetric measurements, the sample was neutralized by adding an appropriate volume of 2 mol L^−1^ NaOH.

A tap water sample was acidified with the addition of 1 mol L^−1^ to a pH of 1.5, and then it was subjected to a UV mineralization process within 3 h. Before the voltammetric analysis, the sample was neutralized by adding an appropriate amount of 2 mol L^−1^ NaOH.

### 2.4. Standard Procedure of Measurement

The electrochemical cell was filled with 0.5 mL of 1 mol L^−1^ acetate buffer (pH 3.3). The solution was brought to a volume of 10 mL by the appropriate amount of deionized water. During the standard measurement procedure, the following potential sequence was applied to the gold microelectrode array. Firstly, based on the previous observations [38,40], the working microelectrode was activated by applying a short negative potential impulse at −2.5 V within 2 s. Secondly, the deposition step was performed at a potential of 0.1 V within 90 s. During this stage of measurement, metallic copper was deposited on the gold microelectrode’s surface. After a 5 s equilibration time, square-wave voltammograms were recorded by scanning the potential from −0.1 to 0.7 V. The operational parameters were as follows: frequency of 250 Hz, amplitude of 25 mV and step potential of 2 mV. The measurements were conducted without prior deoxygenation of the solutions.

## 3. Results

**Figure 1:**
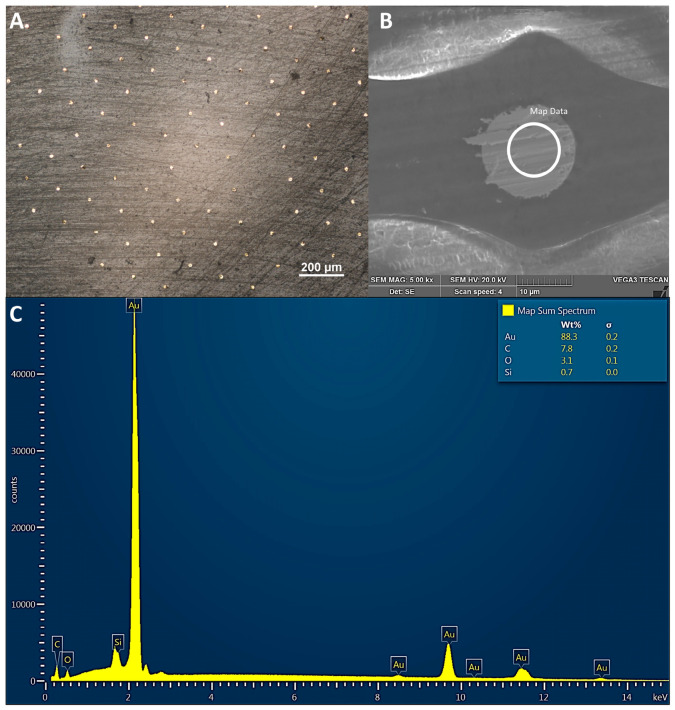
(**A**) An optical image of a part of the surface of the presented gold microelectrode array; (**B**) SEM micrograph of an individual gold microelectrode, with the mapping area used for study EDS spectrum; (**C**) EDS spectrum of the indicated surface of the gold microelectrode array.

In the research presented below, a novel type of solid gold microelectrode array was utilized, featuring an extended lifetime achieved through its unique design. Such a sensor preparation method yields a highly reusable working electrode, in contrast to microelectrodes fabricated via, e.g., lithographic, inkjet-printed or screen-printed techniques [41,42,43]. A real view of the surface of a gold microelectrode array taken with the MA200 Inverted Metallographic Microscope (Nikon, Tokyo, Japan) is presented in Figure 1A. An SEM micrograph of an individual gold microelectrode and an EDS spectrum of the part of the gold microelectrode surface are presented in Figure 1B and Figure 1C, respectively. The results indicated that no metallic elements other than gold were detected on the microelectrode surface. The observed carbon signal originates from residual contaminants within the chamber that accumulated during EDS analysis. Moreover, the presence of Si and C can be attributed to contamination from the sandpaper (SiC) used during polishing, as both elements are primary constituents of the abrasive material. The presence of oxygen is connected with the fact that the preform used for the fabrication of the microelectrode array is made of silica, and it is transferred to the electrode surface as a contaminant during polishing.

### 3.1. Microelectrode Properties Testing

**Figure 2:**
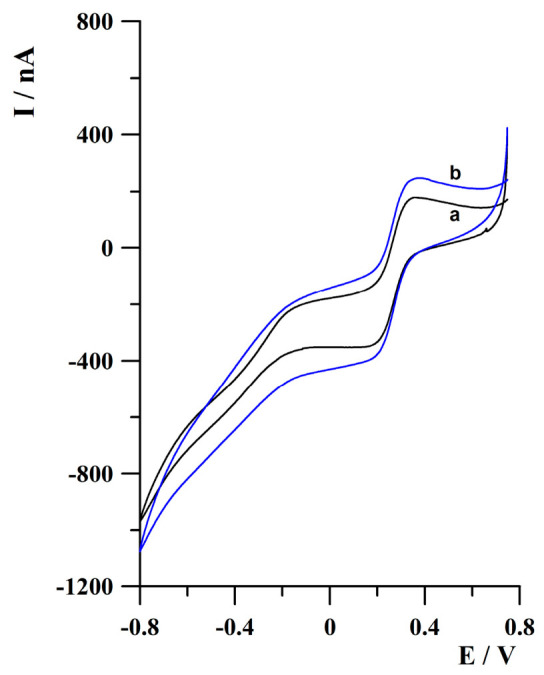
Cyclic voltammogram of 1 × 10^−3^ mol L^−1^ K_3_Fe(CN)_6_ in 1 mol L^−1^ KCl electrolyte. Scan rate: (a) 10; (b) 20 mV s^−1^.

Cyclic voltammetry was employed to investigate the microelectrode characteristics of the developed small-sized electrode. The cyclic voltammetric measurements were conducted from a solution containing 1 × 10^−3^ mol L^−1^ K_3_Fe(CN)_6_ and 1 mol L^−1^ KCl. The obtained cyclic voltammogram is shown in Figure 2. In agreement with the established literature [44], the results suggest that the individual diffusion layers within the array partially overlap. However, the preservation of a sigmoidal voltammetric wave demonstrates that steady-state radial diffusion is maintained at the individual microelectrodes, verifying microelectrode behavior.

### 3.2. Optimization of the Procedure Parameters

#### 3.2.1. Selection of the pH and the Concentration of the Supporting Electrolyte

**Figure 3:**
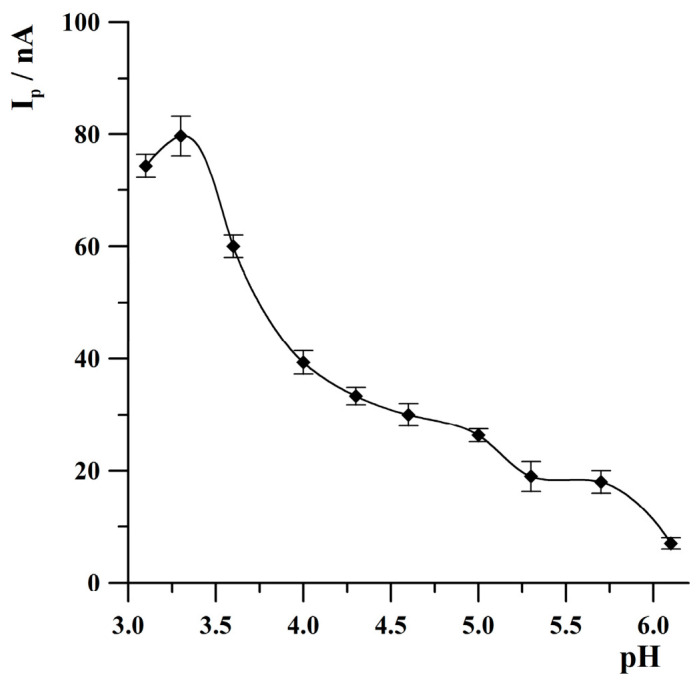
The effect of acetate buffer pH on the Cu(II) analytical signal. Experimental conditions: Cu(II) concentration 2 × 10^−8^ mol L^−1^; deposition potential −0.1 V; deposition time 60 s. The error bars represent the standard deviation (*n* = 3) (unless stated otherwise, *n* represents consecutive measurements repeated with the same microelectrode array and the same solution within one day).

The influence of the acetate buffer pH on the copper analytical signal was investigated across a range of 3.1 to 6.1, with the corresponding results depicted in Figure 3. The Cu(II) concentration was 2 × 10^−8^ mol L^−1^. It was observed that the Cu(II) peak height reached its maximum at pH 3.3, followed by a gradual decline in less acidic media.

Furthermore, the effect of the acetate buffer concentration (pH 3.3) was evaluated between 0.01 and 0.2 mol L^−1^. As illustrated in Figure S1 (Supplementary Material), the Cu(II) peak current attained its highest value within the concentration range of 0.025 to 0.05 mol L^−1^, whereas higher buffer concentrations resulted in an attenuated copper signal.

As a result of this study, the pH and concentration of the acetate buffer of 3.3 and 0.05 mol L^−1^, respectively, were chosen as the optimal values.

#### 3.2.2. Selection of the Activation Step Conditions

**Figure 4:**
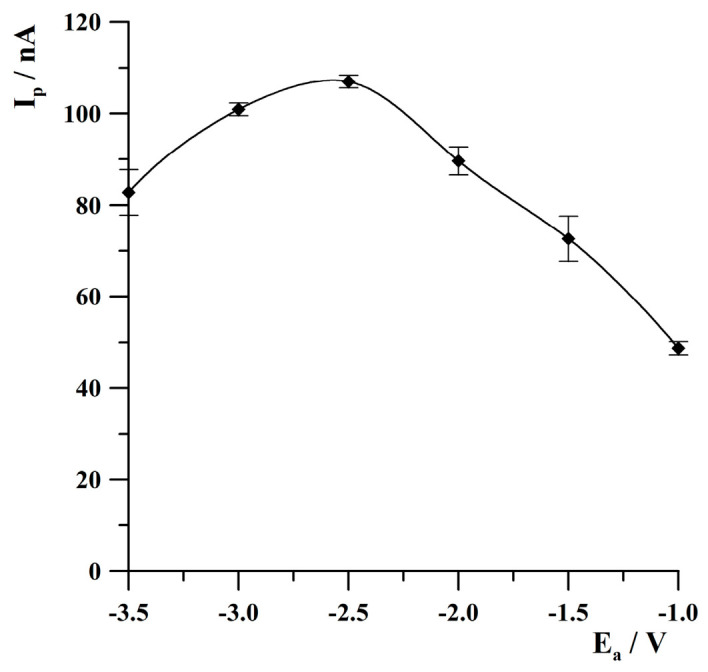
Effect of the activation potential on the Cu(II) analytical signal. Experimental conditions: Cu(II) concentration 2 × 10^−8^ mol L^−1^; deposition potential −0.1 V; deposition time 60 s. The error bars represent the standard deviation (*n* = 3).

The activation step is an important stage of the standard measurement procedure when using solid metal electrodes. During this step, the application of a short potential pulse at a relatively high negative potential facilitates the reduction of any metal oxides, which is frequently addressed in the literature [38,45]. Consequently, such a way of electrode pretreatment ensures that the resulting analytical signals are better shaped and reproducible. Firstly, the activation potential was tested. Throughout these experiments, the Cu(II) concentration was 2 × 10^−8^ mol L^−1^. The influence of the activation potential was investigated within the range of −1.0 to −3.5 V, with the corresponding results presented in Figure 4. The Cu(II) peak current exhibited an increase as the potential changed from −1.0 to −2.5 V, reaching its maximum value at −2.5 V. Beyond this point, further changes toward more negative potentials resulted in a subsequent decrease in the Cu(II) analytical signal, because as the activation potential values become more negative, there is an increase in the intensity of hydrogen evolution. Consequently, the microelectrode surface becomes partially blocked. As an optimal activation potential, the value of −2.5 V was chosen.

Subsequently, the activation time was investigated within the range of 0 to 10 s. The Cu(II) concentration was 2 × 10^−8^ mol L^−1^. The obtained results are presented in Figure S2 in the Supplementary Material. The Cu(II) peak current increased throughout the entire tested time range. An optimal value of 2 s was selected to minimize hydrogen evolution during the activation step.

The further studies were conducted at an activation potential of −2.5 V within 2 s.

#### 3.2.3. Selection of the Deposition Conditions

**Figure 5:**
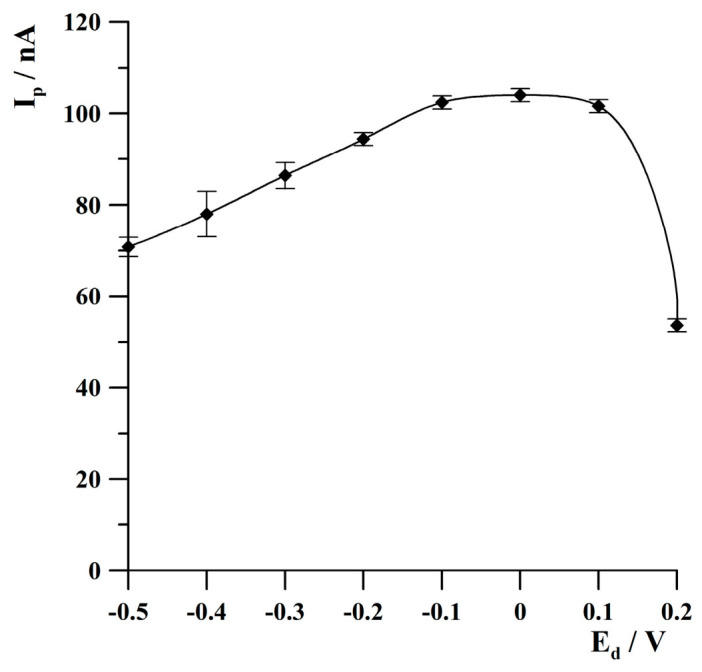
The effect of the deposition potential on the Cu(II) analytical signal. Cu(II) concentration was 2 × 10^−8^ mol L^−1^. Deposition time: 60 s. The error bars represent the standard deviation (*n* = 3).

The influence of the deposition potential on the Cu(II) analytical signal was examined within the range of −0.5 to 0.2 V, using the Cu concentration of 2 × 10^−8^ mol L^−1^ and a deposition time of 60 s. The corresponding results are presented in Figure 5. It was observed that the copper peak current increased marginally from −0.5 to −0.1 V, reached a maximum plateau between −0.1 and 0.1 V, and subsequently decreased as the potential shifted toward more positive anodic values. The deposition potential of 0.1 V was selected as the optimal condition to prevent the possible deposition of foreign ions, e.g., lead, at more negative potential values. Consequently, shifting toward more positive deposition potential values prevents possible interferences, ensuring the determination of Cu(II) ions even in matrices containing an excess of Pb(II) ions.

**Figure 6:**
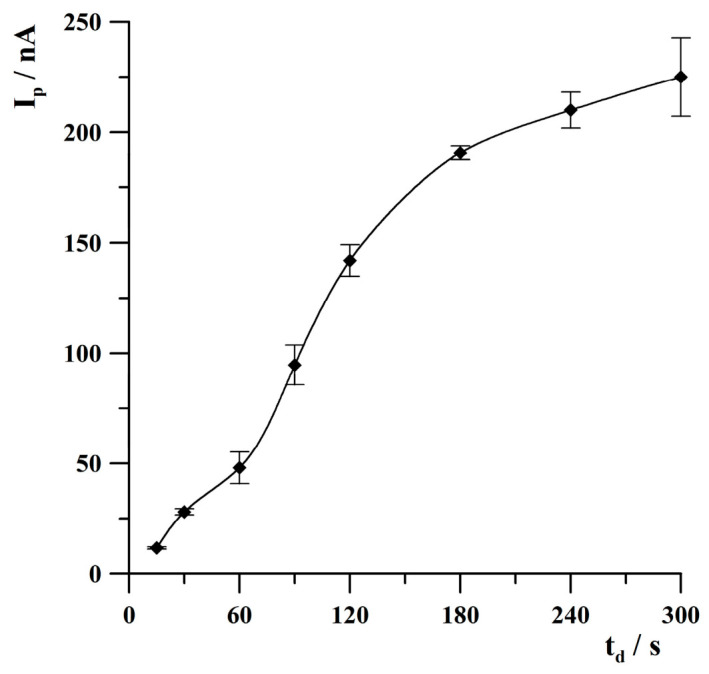
Effect of deposition time on the Cu(II) analytical signal. Experimental conditions: Cu(II) concentration 1 × 10^−8^ mol L^−1^, deposition potential: 0.1 V. The error bars represent the standard deviation (*n* = 3).

The influence of the deposition time on the analytical signal of Cu(II) ions was evaluated across a range of 15 to 300 s, using the Cu(II) concentration of 1 × 10^−8^ mol L^−1^. The corresponding results are depicted in Figure 6. It was found that the copper peak height increased in the whole tested range; however, the observed increase exhibited non-linear behavior in the range from 180 to 300 s. The slower growth of the copper signal at extended deposition periods can be attributed to the formation of multilayer coverage at the microelectrode surface. Further studies were conducted mainly at 90 s of deposition, as this value represents an optimal compromise, ensuring high sensitivity while maintaining a short analysis time and avoiding the non-linear effects associated with longer deposition periods.

#### 3.2.4. Selection of the Square Wave Parameters

**Figure 7:**
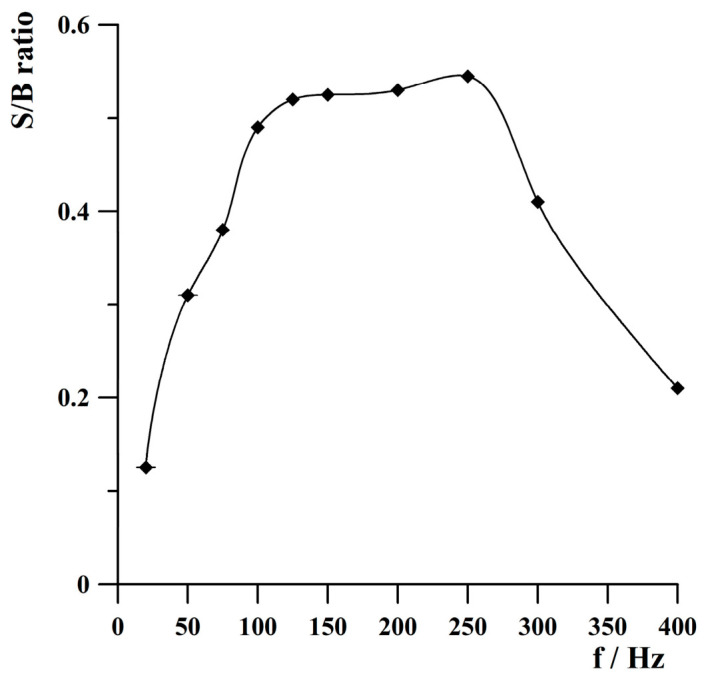
Dependence of the copper signal-to-background ratio on the square-wave frequency. Experimental conditions: Cu(II) concentration 1 × 10^−8^ mol L^−1^, deposition potential 0.1 V, deposition time 90 s.

Initially, the effect of frequency on the copper analytical signal was investigated across the range of 20 to 400 Hz. Cu(II) concentration was 1 × 10^−8^ mol L^−1^, with the amplitude and step potential maintained at 50 mV and 2 mV, respectively. The corresponding results are depicted in Figure 7 and Figure S3 (Supplementary Material). Although the copper peak height exhibited a continuous increase throughout the evaluated frequency range, the most favorable signal-to-background ratio was achieved at 250 Hz. Consequently, this frequency was selected for subsequent experiments.

The effect of amplitude on the copper peak current was evaluated within the range of 10 to 100 mV while maintaining the frequency and step potential at 250 Hz and 2 mV, respectively. Cu(II) concentration was 1 × 10^−8^ mol L^−1^. The results of this study are presented in Figure S4 (Supplementary Material). The optimal amplitude value of 25 mV was chosen because under this condition the most favorable value of the signal-to-background ratio was observed.

The influence of the step potential on the copper analytical signal was evaluated across the range of 1 to 10 mV while maintaining frequency and amplitude at 250 Hz and 25 mV, respectively. Cu(II) concentration was 1 × 10^−8^ mol L^−1^. The corresponding results are compiled in Figure S5 (Supplementary Material). The most favorable copper signal-to-background ratio was recorded at a step potential of 2 mV, which was consequently selected for subsequent experiments.

### 3.3. Calibration Studies

The calibration studies were performed for the deposition times of 30 and 90 s to broaden the linear dynamic range while maintaining a short analysis time. These periods were chosen because the analytical signal increases linearly with deposition time up to 120 s, thereby ensuring reliable determination within different concentration ranges.

For the deposition time of 30 s, the calibration graph for Cu(II) exhibited linearity across the concentration range of 2 × 10^−9^ to 2 × 10^−7^ mol L^−1^. The corresponding linear regression equation was expressed as y = 5.66x + 7.79, where y and x represent the peak current (in nA) and the Cu(II) concentration (in nmol L^−1^), respectively. The linear correlation coefficient R^2^ was 0.998. The relative standard deviation evaluated at a concentration of Cu(II) of 2 × 10^−8^ mol L^−1^ was 4.5% (*n* = 3).

**Figure 8:**
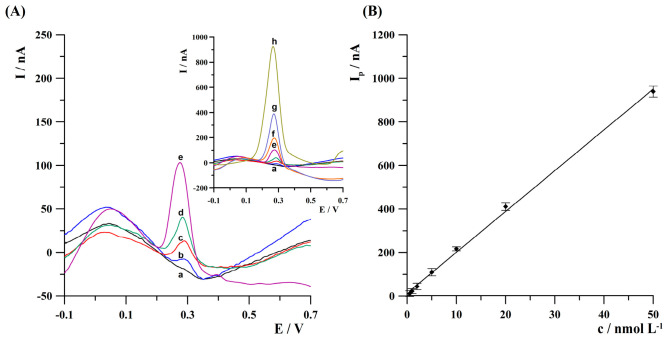
Anodic stripping voltammograms recorded for increasing Cu(II) concentration (**A**) and the corresponding linear calibration graph (**B**). Concentration of Cu(II): (a) 0; (b) 5 × 10^−10^; (c) 1 × 10^−9^; (d) 2 × 10^−9^; (e) 5 × 10^−9^; (f) 1 × 10^−8^; (g) 2 × 10^−8^; (h) 5 × 10^−8^ mol L^−1^. Deposition conditions: 0.1 V, 90 s. The error bars represent the standard deviation (*n* = 3).

For a prolonged deposition time (t_d_ = 90 s), the calibration plot exhibited linearity across the Cu(II) concentration range of 5 × 10^−10^ to 5 × 10^−8^ mol L^−1^. The corresponding linear regression equation was y = 18.8x + 11.2. The linear correlation coefficient R^2^ was 0.998. The relative standard deviation for Cu(II) concentration of 1 × 10^−8^ mol L^−1^ was 4.7% (*n* = 3). The limit of detection calculated based on three times the standard deviation of the intercept divided by the slope of the calibration graph was found to be 1.93 × 10^−10^ mol L^−1^. The representative voltammograms recorded for increasing Cu(II) concentrations and the resulting calibration graph are presented in Figure 8A and Figure 8B, respectively. The analytical characteristics of the developed voltammetric procedure for copper ion determination for deposition times of 30 s and 90 s are presented in Table 2.

### 3.4. Repeatability, Reproducibility and Stability Studies

The repeatability of the analytical signal was evaluated using a solid gold microelectrode array for Cu(II) determination. To assess intra-day precision, peak heights obtained from ten consecutive measurements of a single solution containing the supporting electrolyte and 2 × 10^−8^ mol L^−1^ Cu(II) were compared. The deposition time was 30 s. The calculated relative standard deviation (RSD) was 4.2%. The voltammograms recorded during this study (presented in Figure S6 in the Supplementary Material) confirm the satisfactory repeatability of the copper(II) determination.

Subsequently, inter-day repeatability was evaluated over a five-day period using independently prepared solutions containing 2 × 10^−8^ mol L^−1^ Cu(II), yielding an RSD of 5.8% (*n* = 5). Based on these RSD values, the repeatability of the proposed electroanalytical procedure was deemed satisfactory. The higher RSD value in this case stems from changes in surface morphology resulting from the polishing process.

Furthermore, the reproducibility of the copper(II) analytical signal was investigated by comparing the analytical signals obtained from five distinct gold microelectrode arrays, fabricated according to the protocol described in Section 2.1. The voltammetric responses were recorded in a supporting electrolyte solution containing 2 × 10^−8^ mol L^−1^ Cu(II). The resulting RSD calculated from these measurements was 5.3% (*n* = 5), confirming satisfactory reproducibility.

Finally, the long-term stability of the proposed gold microelectrode array was monitored. The sensor stability, evaluated based on the reproducibility of the copper(II) analytical signal, was assessed in a supporting electrolyte containing 2 × 10^−8^ mol L^−1^ Cu(II) by performing five consecutive measurements approximately three months after the initial procedure development. The resulting RSD value was 4.0%, confirming the excellent long-term stability of the developed microelectrode array.

### 3.5. Interference Effects

To evaluate the selectivity of the developed method for natural water analysis, the potential interferences were investigated using a solution containing Cu(II) at a concentration of 2 × 10^−8^ mol L^−1^, under optimized deposition conditions of 0.1 V within 30 s. As summarized in Table 3, the majority of the tested foreign ions did not significantly alter the Cu peak current, even when present in a high excess. The occurrence of potential interferences was also investigated in the presence of Na_2_EDTA as a model of a complexing agent. It was found that in the presence of 1 × 10^−4^ and 2 × 10^−4^ mol L^−1^ of Na_2_EDTA, the Cu peak current decreased to 97 and 84%, respectively.

Furthermore, the influence of the organic matter on the copper(II) peak current has been tested. It was observed that the presence of humic acids at concentrations of 2, 5 and 10 mg L^−1^ caused a decrease of the copper analytical signal to 96.8, 87.2 and 61.3% of its original value, respectively. The presence of fulvic acids at a concentration of 2, 5 and 10 mg L^−1^ decreased the copper peak current to 95.9, 84.2 and 58.3% of its primary value, respectively. Based on these observations, it can be concluded that the procedure can be used for the determination of labile forms of copper; however, after mineralization of the environmental samples, the total copper content in the sample can be determined. The observed impact of Na_2_EDTA and organic matter leads to the conclusion that mineralization of the sample is recommended if the total copper concentration is determined.

Based on the comprehensive interference data, it can be concluded that the developed voltammetric procedure displays satisfactory selectivity toward Cu(II) ions.

### 3.6. Analysis of the Certified Reference Material and Real Water Samples

**Figure 9:**
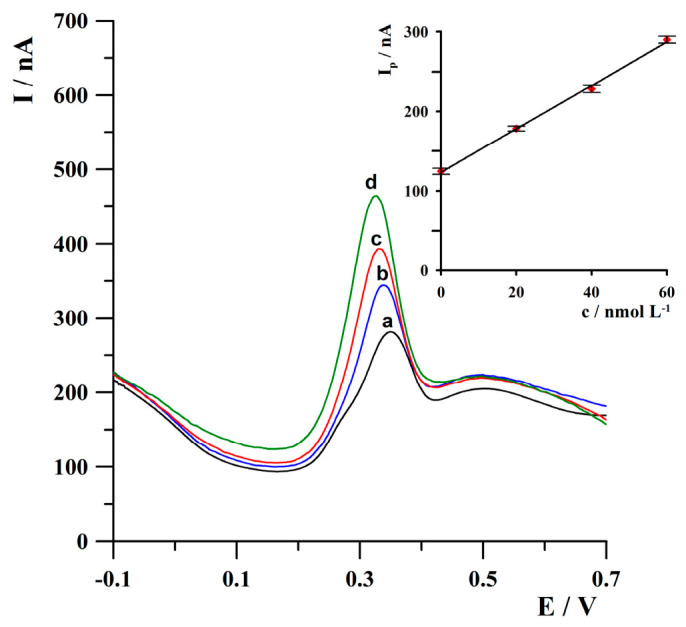
Anodic stripping voltammograms recorded during analysis of the certified water reference material: (a) supporting electrolyte with a sample of TM 26.5; (b) as (a) + 2 × 10^−8^ mol L^−1^; (c) as (b) + 2 × 10^−8^ mol L^−1^; (d) as (c) + 2 × 10^−8^ mol L^−1^ of Cu(II). Deposition conditions: 0.1 V, 30 s. Inset: Corresponding standard addition graph. The error bars represent the standard deviation (*n* = 3).

The accuracy of the developed analytical procedure was verified by analyzing the certified water reference material TM 26.5 (lake water). Prior to analysis, the reference material was diluted fivefold and neutralized with an appropriate volume of 2 mol L^−1^ NaOH. The standard addition graph was expressed by the equation y = 2.73x + 123.3, where x is the Cu(II) concentration in nmol L^−1^, whereas y is the peak current in nA. The linear correlation coefficient R^2^ was 0.998. The determination of Cu(II) was performed using the standard addition method with a deposition time of 30 s. The certified Cu(II) content in this reference material is 14.1 ± 1.0 µg L^−1^, whereas the determined concentration was 14.3 ± 0.78 µg L^−1^. The obtained results of the certified reference material analysis confirmed the accuracy of the developed voltammetric procedure for the analysis of natural water samples. The voltammograms obtained during the validation studies and the corresponding standard addition graph (in the form of an inset) are presented in Figure 9.

**Figure 10:**
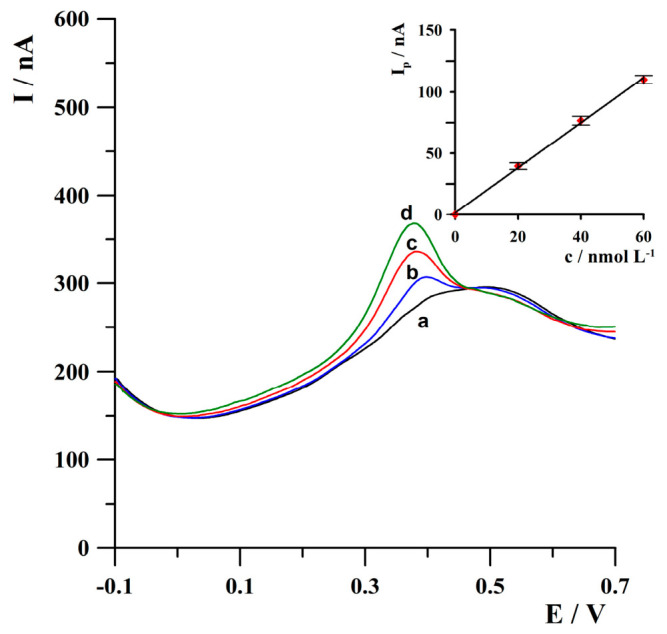
Anodic stripping voltammograms recorded during analysis of the environmental water sample: (a) analyzed river water sample (dilution factor: 2); (b) as (a) + 2 × 10^−8^ mol L^−1^; (c) as (b) + 2 × 10^−8^ mol L^−1^; (d) as (c) + 2 × 10^−8^ mol L^−1^ of Cu(II). Deposition conditions: 0.1 V, 30 s. Inset: the corresponding standard addition graph. The error bars represent the standard deviation (*n* = 3).

The subsequent objective of this research was the practical application of the developed procedure for analysis of the environmental water samples collected from the Bystrzyca River (Eastern Poland). Following the sample preparation protocol described in Section 2.3, a 5 mL aliquot of the sample was transferred to the electrochemical cell (resulting in a twofold dilution), and voltammetric measurements were carried out using the standard addition method. The voltammograms recorded for the Bystrzyca River water sample did not exhibit a detectable analytical signal for Cu(II), indicating that the copper(II) concentration was below the detection limit of the proposed procedure. Therefore, recovery studies were performed by spiking the analyzed samples with known Cu(II) concentrations to verify the reliability of the method. Three replicate determinations of a sample spiked with successive additions of 2 × 10^−8^ mol L^−1^ Cu(II) yielded average recovery values ranging from 98.5 to 103.6%, with a relative standard deviation of 3.3%. The voltammograms recorded during this analysis are presented in Figure 10. These results confirm that the developed procedure is suitable for Cu(II) determination in real water samples.

**Figure 11:**
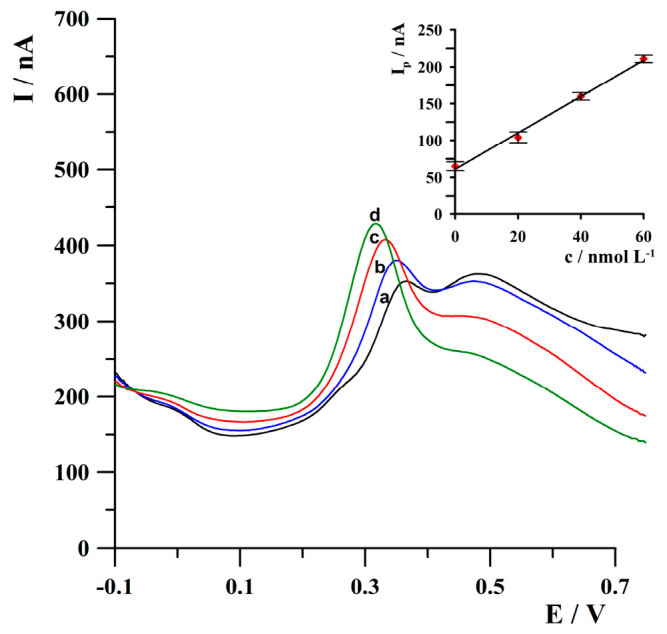
Anodic stripping voltammograms recorded during analysis of a tap water sample: (a) analyzed sample (dilution factor: 5); (b) as (a) + 2 × 10^−8^ mol L^−1^; (c) as (b) + 2 × 10^−8^ mol L^−1^; (d) as (c) + 2 × 10^−8^ mol L^−1^ of Cu(II). Deposition conditions: 0.1 V, 30 s. Inset: the corresponding standard addition graph. The error bars represent the standard deviation (*n* = 3).

Moreover, the developed voltammetric procedure was used for analyzing a tap water sample. Before the proper analysis, the sample was prepared according to the procedure described in Section 2.3. A total of 2 mL of such a prepared sample was transferred to the voltammetric cell (factor of dilution was 5), and the determination of Cu(II) was performed using the standard addition method, following the developed procedure. The determined Cu(II) concentration in the tap water sample was equal to 7.85 ± 0.95 µg L^−1^. This value is in agreement with the result equal to 7.61 ± 0.13 µg L^−1^, obtained using ICP-MS as a comparative method. The voltammograms recorded during the determination of Cu(II) in a tap water sample are presented in Figure 11.

To conclude, the results obtained for the analysis of the certified water reference material, river water and tap water samples confirm that the developed voltammetric procedure is suitable for Cu(II) determination in real water samples.

## 4. Conclusions

The presented study demonstrates the application of a long-term use and environmentally friendly solid gold microelectrode array for the simple, fast, sensitive and selective labile and total copper(II) determination by anodic stripping voltammetry. The procedure does not necessitate any modification of the electrode surface, thus facilitating the composition of the supporting electrolyte and the measurement procedure itself, and is therefore in line with the principles of green chemistry.

The presented voltammetric procedure offers a wide, two orders of magnitude linear dynamic range from 5 × 10^−10^ to 5 × 10^−8^ mol L^−1^ for a deposition time of 90 s. The obtained detection limit of Cu(II) determination was 1.93 × 10^−10^ mol L^−1^. The determination of Cu(II) in the water certified reference material yielded results consistent with certified values, confirming the procedure’s accuracy for the analysis of analogous environmental matrices. The developed procedure was also used for Cu(II) determination in a tap water sample, obtaining satisfactory agreement with the results of the analysis performed using the ICP-MS as a comparative method. In addition, the analysis of environmental water samples yielded excellent recovery values, demonstrating the reliability of the proposed voltammetric procedure.

## Figures and Tables

**Table 1:** Comparative evaluation of analytical parameters for stripping voltammetric determination of copper(II) on diverse working electrodes.

Working Electrode	Preconcentration Time [s]	Linear Range [nmol L^−1^]	Detection Limit [nmol L^−1^]	Comments	Ref.
HMDE	90	3.15–1575	1.57	AdSV; analysis of commercial salts and aluminum alloys	[12]
MFE	300	20–400	2.4	ASV; analysis of gasoline samples	[15]
Au microwire	60	4.7–126	0.8	ASV; determination of dissolved copper species in seawater	[16]
Modified AuE	1500	800–100,000	400	CV; electrode modified with penicillamine; analysis of tap water	[17]
Modified AuE	420	0.1–100 100–100,000	0.1	CV; electrode modified with N-acetyl-L-cysteine; analysis of a reference material and lake water	[18]
AuE	90	79–1575	4.9	ASV; simultaneous determination of Cu(II) and tert-butylhydroquinone in biodiesel	[19]
AuµE	210	1–90	0.3	ASV; tap water, lake water and commercial drinking water samples	[20]
Modified pencil graphite electrode	60	2 × 10^−4^–0.05	4.8 × 10^−5^	ASV; electrode modified with D-penicillamine functionalized nano-cellulose; analysis of tap and river water samples	[22]
Modified carbon paste electrode	180 600	10–1000 1–10	0.5	ASV; electrode modified with N-phenylcinnamohydroxamic acid; analysis of potable water and commercial lithium chloride	[24]
Modified carbon paste electrode	900	50–5000	15	CSV; electrode modified with natural zeolite; analysis of commercial dried tomato and Bakosel capsule	[25]
Modified carbon nanotube paste electrode	300	79–16,000	10	ASV; electrode modified with crosslinked chitosan; analysis of industrial wastewater, natural water and human urine samples	[26]
BiF/GCE	120	126–317	31	ASV; rotating electrode; analysis of surface river water samples	[30]
SbF/SPCE	120	83–1570	25	ASV; analysis of a groundwater certified reference material	[31]
SbF/CPE	120	up to 1890	22.8	ASV; analysis of natural water samples	[32]
PtNP/CPE	600	39–1600	3.9	ASV; analysis of certified reference materials	[34]
Modified GCE	90	157–1102	1.73	ASV; electrode modified with ferrocenyl crown ether; analysis of a mining effluent and urine samples	[35]
Sb_2_O_3_/CNTPE	90	31.5–1575	6.1	ASV; analysis of tap water sample	[36]
Ir-UMEA	180	315–1575	78.7	ASV; microlithographically fabricated electrode; analysis of tap water and bottled drinking water samples	[37]
AuµE array	30 90	2–200 0.5–50	0.81 0.193	ASV; analysis of natural water sample and certified reference material	[this work]

Symbols explanation: AdSV—adsorptive stripping voltammetry; ASV—anodic stripping voltammetry; CV—cyclic voltammetry; CSV—cathodic stripping voltammetry; AuE—gold electrode; AuµE—gold microelectrode; BiF/GCE—bismuth film plated on glassy carbon substrate; SbF/SPCE—antimony film plated on screen-printed carbon electrode; SbF/CPE—antimony film carbon paste electrode; PtNP/CPE—carbon paste electrode modified with platinum nanoparticles; GCE—glassy carbon electrode; Sb_2_O_3_/CNTPE—an antimony trioxide-modified multi-walled carbon nanotube paste electrode; Ir-UMEA—array of iridium ultramicroelectrodes.

**Table 2:** The analytical performance of the developed voltammetric procedure for copper ion determination for deposition times of 30 s and 90 s.

Deposition Time [s]	Slope [nA/nmol L^−1^] ± SD	Intercept [nA] ± SD	Correlation Coefficient	Linear Range [nmol L^−1^]	Limit of Detection [nmol L^−1^] *
30	5.66 ± 0.11	7.79 ± 1.39	0.998	2–200	0.81
90	18.8 ± 0.31	11.2 ± 1.14	0.998	0.5–50	0.193

* The value of detection limits was calculated using the LINES function in MS Excel 2010.

**Table 3:** Relative analytical signal of Cu(II) in the presence and absence of various interfering ions. The concentration of Cu(II) was 2 × 10^−8^ mol L^−1^.

Interfering Ion	A Molar Excess of the Interfering Ion	^1^ Relative Signal of Cu(II) ± SD
Mn(II)	100	0.95 ± 0.036
Co(II)	100	1.08 ± 0.043
Ni(II)	100	1.04 ± 0.041
Zn(II)	100	0.97 ± 0.042
Fe(III)	100	1.04 ± 0.037
Ti(IV)	100	1.02 ± 0.043
Pb(II)	50 100	0.94 ± 0.042 0.78 ± 0.041
Hg(II)	5 10	1.05 ± 0.0 43 1.34 ± 0.045
As(III)	20 50	0.95 ± 0.039 0.81 ± 0.046
K(I)	250,000	0.97 ± 0.042
Mg(II)	250,000	0.98 ± 0.039
Ca(II)	250,000	1.03 ± 0.038
Cl^−^	250,000	0.96 ± 0.041
SO_4_^2−^	250,000	0.95 ± 0.038

^1^ Relative signal–peak current ratio in the presence and absence of the molar excess of an interferent.

## Data Availability

Data is contained within the article or Supplementary Materials.

## Supplementary Materials

The following supporting information can be downloaded at: https://www.mdpi.com/article/10.3390/s26134305/s1, Figure S1. The effect of the acetate buffer concentration on Cu(II) analytical signal. Cu(II) concentration was 2 × 10^−8^ mol L^−1^. Deposition conditions: −0.1 V, 60 s. Figure S2. The effect of the activation time on the Cu(II) analytical signal. Cu(II) concentration was 2 × 10^−8^ mol L^−1^. Deposition conditions: −0.1 V, 60 s. Figure S3. The effect of the frequency on the Cu(II) analytical signal. Cu(II) concentration was 1 × 10^−8^ mol L^−1^. Deposition conditions: 0.1 V, 90 s. Figure S4. Copper signal-to-background ratio as a function of amplitude. Cu(II) concentration was 1 × 10^−8^ mol L^−1^. Deposition conditions: 0.1 V, 90 s. Figure S5. Copper signal-to-background ratio as a function of the step potential. Cu(II) concentration was 1 × 10^−8^ mol L^−1^. Deposition conditions: 0.1 V, 90 s. Figure S6. Anodic stripping voltammograms of copper(II) obtained for ten subsequent measurements conducted from the same solution during repeatability studies. Cu(II) concentration was 2 × 10^−8^ mol L^−1^. Conditions of deposition: 0.1 V, 30 s.
