# Supplementary material for: Simple, Fast and Sensitive Voltammetric Procedure for Copper Ion Determination Using a Solid Gold Microelectrode Array
Source: Sensors (Basel). 2026 Jul 7;26(13):4305. doi: 10.3390/s26134305 (PMC13363986; doi:10.3390/s26134305)
Supplement: Supplementary file 1 [file sensors-26-04305-s001.zip › sensors-4360221-supplementary.pdf]

Supplementary Material

Simple, fast and sensitive voltametric procedure for copper ions determination using a gold microelectrode array

Mieczyslaw Korolczuk, Mateusz Ochab and Iwona Gęca*

Institute of Chemical Sciences, Faculty of Chemistry, Maria Curie Sklodowska University, 20-031 Lublin, Poland; mieczyslaw.korolczuk@mail.umcs.pl (M.K.); mateusz.ochab@mail.umcs.pl (M.O.)

*correspondence: iwona.geca@mail.umcs.pl

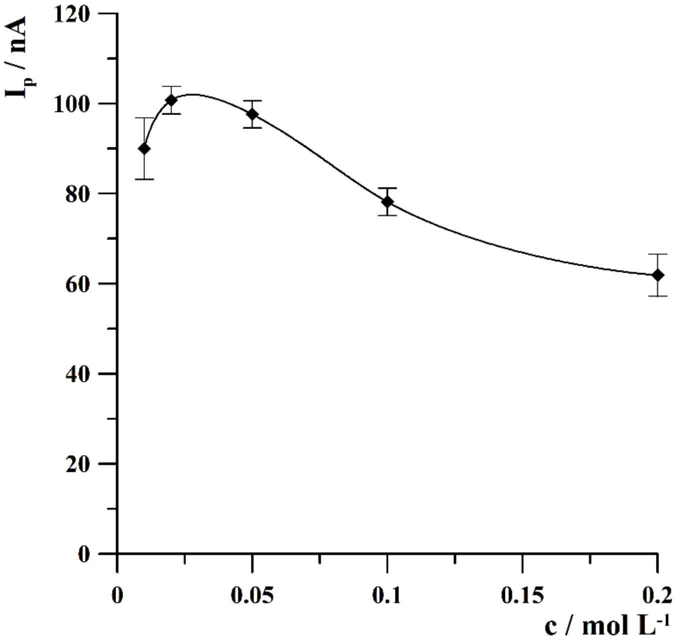

Figure S1. The effect of the acetate buffer concentration on Cu(II) analytical signal. Cu(II) concentration was 2×10^{-8} mol L^{-1}. Deposition conditions: −0.1 V, 60 s.

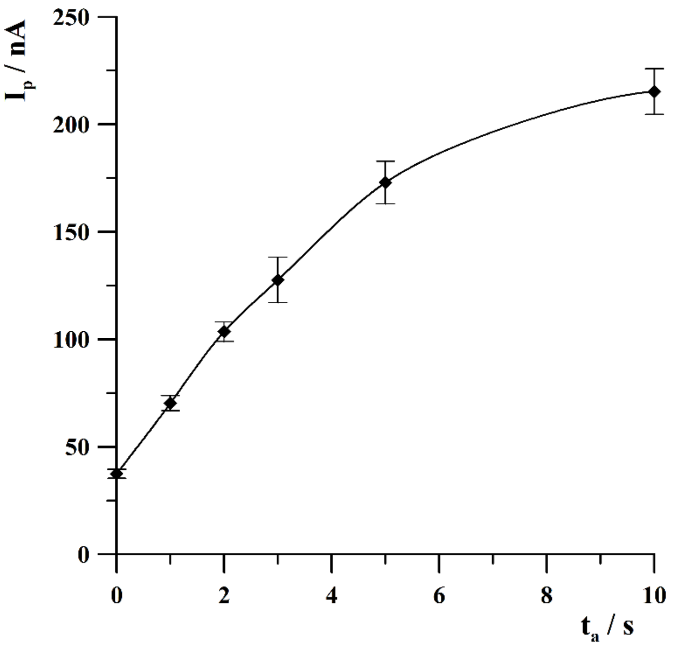

Figure S2. The effect of the activation time on the Cu(II) analytical signal. Cu(II) concentration was 2×10^{-8} mol L^{-1}. Deposition conditions: −0.1 V, 60 s.

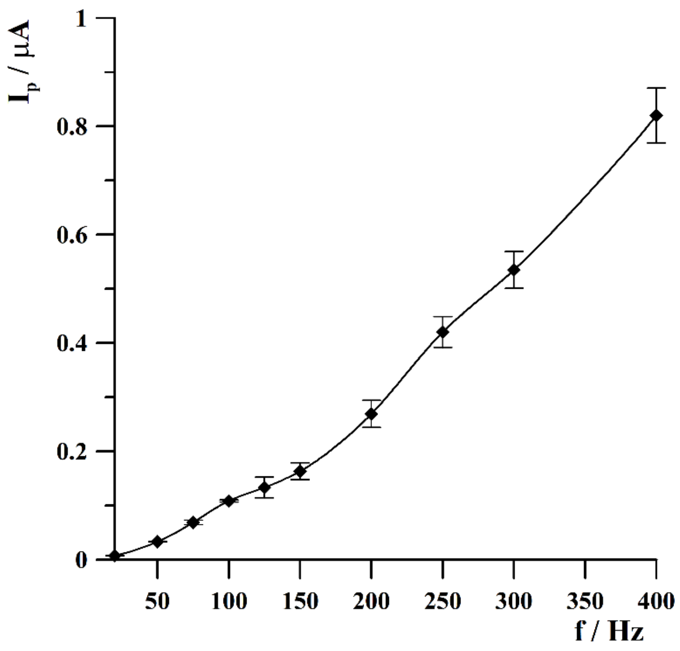

Figure S3. The effect of the frequency on the Cu(II) analytical signal. Cu(II) concentration was 1×10^{-8} mol L^{-1}. Deposition conditions: 0.1 V, 90 s.

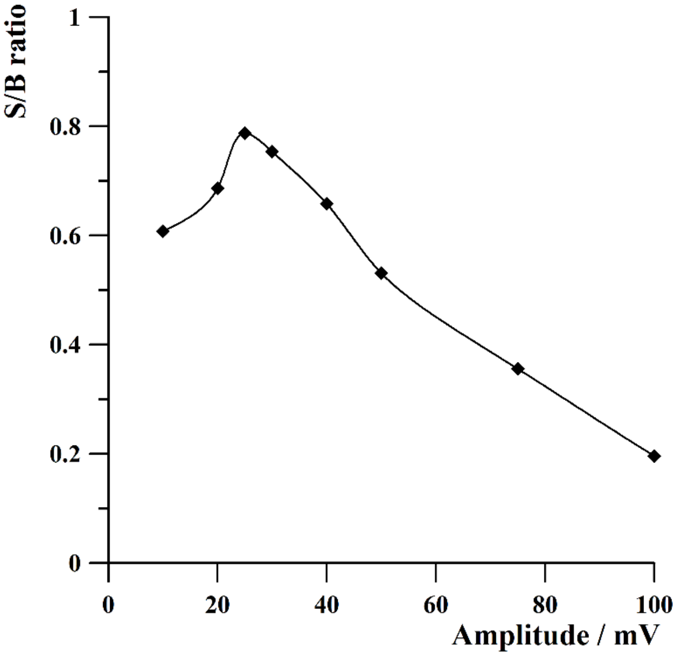

Figure S4. Copper signal-to-background ratio as a function of amplitude. Cu(II) concentration was 1 × 10^{-8} mol L^{-1}. Deposition conditions: 0.1 V, 90 s.

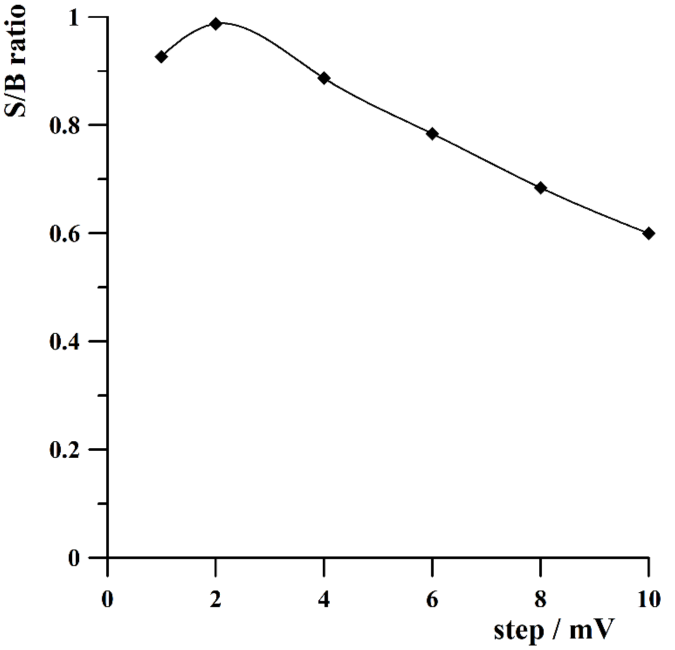

Figure S5. Copper signal-to-background ratio as a function of the step potential. Cu(II) concentration was 1 × 10^{-8} mol L^{-1}. Deposition conditions: 0.1 V, 90 s.

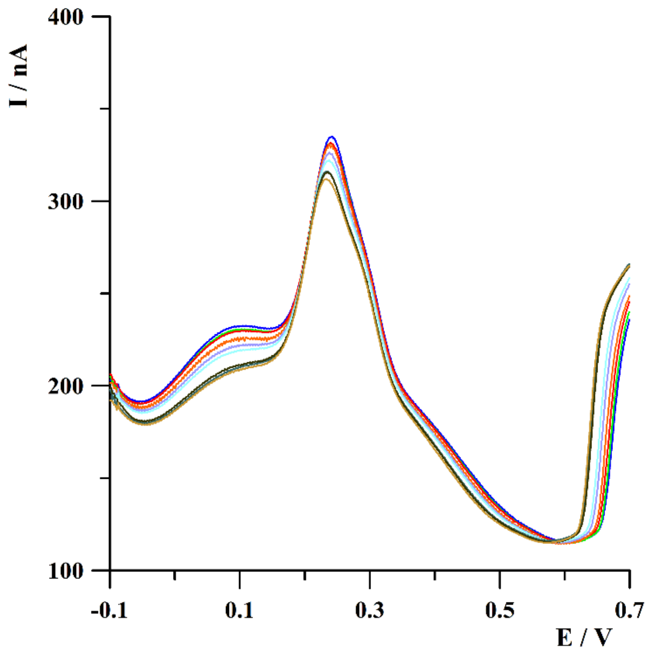

Figure S6. Anodic stripping voltammograms of copper(II) obtained for ten subsequent measurements conducted from the same solution during repeatability studies. Cu(II) concentration was 2×10^{-8} mol L^{-1}. Conditions of deposition: 0.1 V, 30 s.
